# Epidemiological patterns of Crimean-Congo hemorrhagic fever in Iran and its neighboring endemic high-risk territories: A systematic review

**DOI:** 10.1016/j.parepi.2026.e00499

**Published:** 2026-03-26

**Authors:** Jalal Mohammadi, Mohammad Amin Rezaei, Kourosh Azizi, Zahra Nasiri, Mohsen Kalantari

**Affiliations:** a Marvdasht Persian Gulf Health Center, Deputy of Health and Medical Affairs, Shiraz University of Medical Sciences, Shiraz, Iran; b Department of Foreign Language, Shiraz Branch, Islamic Azad University, Shiraz, Iran; c Research Center for Health Sciences, Institute of Health, Department of Vector Biology and Control of Diseases, School of Health, Shiraz University of Medical Sciences, Shiraz, Iran

**Keywords:** Crimean Congo hemorrhagic fever, Epidemiology, Iran, Systematic review

## Abstract

This study synthesizes epidemiological, virological, and ecological data from Iran and its neighboring endemic countries between 2005 and 2025. Analysis of 55 included studies revealed that the disease disproportionately affects males aged 15–44, particularly livestock handlers, slaughterhouse workers, and housewives, with elevated incidence in border provinces during spring and summer. *Hyalomma* ticks were identified as the primary reservoirs and vectors, while domestic ruminants—sheep, goats, and cattle —were confirmed as served as amplifying hosts. Phylogenetic investigations highlighted the Asia 1 genotype (Clade IV) across all three nations. Case fatality rates varied regionally, ranging from 8% to 20% in Iran and escalating to 12%–40% in Iran's territories, reflecting disparities in healthcare access and outbreak management. Endemic transmission hubs were concentrated in interconnected border zones, driven by unregulated livestock trade and seasonal migration that facilitate bidirectional pathogen exchange. The findings underscore the role of ecological connectivity and socioeconomic practices in perpetuating CCHF's regional burden. This review provides a novel, integrated perspective on cross-border transmission dynamics, offering insights for targeted interventions. Addressing these gaps will be critical to curbing CCHF's impact high-risk regions.

## 1 Introduction

Crimean-Congo hemorrhagic fever (CCHF), caused by the *Orthonairovirus haemorrhagiae* (family Nairoviridae), is a severe zoonotic disease transmitted primarily by *Hyalomma* ticks and through contact with infected animal tissues or human bodily fluids (Celina et al., 2025). In this cycle, *Hyalomma* ticks act as both the vector and the primary reservoir, maintaining the virus through transstadial and transovarial transmission. Domestic ruminants, such as sheep, goats, and cattle, develop a transient viremia and serve as amplifying hosts, bridging the virus from infected ticks to humans through direct contact with infected blood or tissues (Korany and Abdelgayed, 2021; Maqbool et al., 2022).

With a case fatality rate (CFR) of up to 50%, CCHF poses a significant public health threat in endemic regions of Asia, Africa, Eastern and Western Europe, and the Middle East (Aslam et al., 2023; Aslani et al., 2017; Shahhosseini et al., 2021). The broader Middle East/West Asia region,^1^ in particular, has emerged as a hotspot for CCHF due to ecological conditions favoring tick proliferation, extensive livestock trade, and human migration patterns (El Ghassem et al., 2023; Ilboudo et al., 2025; Nasiri et al., 2025; Telmadarraiy et al., 2012). Iran, along with Turkey, Iraq, Afghanistan, and Pakistan, represents a high-risk zone characterized by shared ecological corridors, pastoral economies, and frequent cross-border movements that amplify transmission risks (Karimi et al., 2021; Sedaghat et al., 2017; Parhizgari et al., 2017). This systematic review aims to synthesize two decades of data to elucidate the interconnected transmission dynamics across these countries, providing a novel contribution by focusing explicitly on the epidemiological corridors linking Iran with its immediate neighbors, an aspect not comprehensively covered in previous reviews.

## 2 CCHF in Iran and neighboring endemic countries: a regional overview

Since 2005, this region has witnessed recurrent CCHF outbreaks, with Iran, Turkey, Iraq, Afghanistan, and Pakistan reporting the highest burden. These countries share ecological features such as arid and semi-arid climates, pastoral economies, and cross-border livestock movement, creating interconnected transmission networks (Al Salihi et al., 2023; Moradi et al., 2019; Mostafavi et al., 2021).

### 2.1 Iran: a regional epicenter

Iran has been a focal point of CCHF since the first confirmed case in 1999, with over 1500 cases and 200 deaths reported between 2000 and 2021 (Ahmadkhani et al., 2018; World Health Organization, 2024). The disease is endemic in 23 of Iran's 31 provinces, with Sistan and Baluchestan, Golestan, and Kerman accounting for 65% of cases (Biglari et al., 2016; Champour et al., 2016). Occupational exposure remains a key driver: 41.9% of cases occur among ranchers, butchers, and slaughterhouse workers, while housewives represent 20% of cases due to contact with livestock during husbandry or slaughtering practices. Furthermore, seasonal peaks align with *Hyalomma* activity, with 86% of cases reported between March and August (Chinikar et al., 2008; Champour et al., 2014; Chinikar et al., 2016).

Iran's eastern and southeastern provinces, particularly Sistan and Baluchestan bordering Afghanistan and Pakistan, are high-risk zones characterized by intense, unregulated cross-border livestock trade and population movement, which create interconnected transmission networks with neighboring endemic areas (Chinikar et al., 2016).

A 2020 study documented a CFR of 14.5% in this region, attributed to delayed hospitalization and limited access to ribavirin in rural areas (Karim, 2020). The heightened outbreak activity in Sistan and Baluchestan, compared to northeastern provinces bordering Afghanistan, is likely multifactorial. The southeastern region has a longer history of endemicity and more robust surveillance systems, leading to higher case detection. Ecologically, the terrain and climate in Sistan and Baluchestan may be more favorable for *Hyalomma* tick proliferation compared to the more arid deserts and mountains of the northeast. Furthermore, the volume and nature of unregulated cross-border livestock trade with Pakistan through established informal routes in the southeast are a particularly intense driver of viral introduction, which may be less pronounced on the northeastern border with Afghanistan (Karim, 2020).

While the efficacy of ribavirin against CCHFV remains debated, its potential benefit is thought to be greatest when administered early in the course of the disease. The limited healthcare infrastructure in these remote regions often prevents at-risk individuals from receiving prompt diagnosis and any potential antiviral therapy, which likely contributes to the elevated CFR (Chinikar et al., 2012). Phylogenetic analyses reveal that 80% of Iranian CCHF virus (CCHFV) strains belong to the Asia 1 genotype (Clade IV), closely related to strains circulating in Afghanistan and Pakistan, suggesting a shared ecological niche and historical viral dispersal, potentially facilitated by cross-border livestock movement (Chinikar et al., 2004). Despite nationwide surveillance programs initiated in 2000, challenges persist, including underreporting in conflict-affected border regions and resistance to acaricides in tick populations (Choubdar et al., 2019).

Notably, while western provinces of Iran (such as Ilam and Kermanshah) share ecological features with endemic areas of Iraq and harbor competent *Hyalomma* vectors, reported CCHF cases in these regions remain sparse (Telmadarraiy et al., 2010). This discrepancy may be attributed to several factors, including disparities in surveillance intensity, which is historically focused on known eastern hotspots, potentially leading to under-diagnosis of sporadic cases in the west. Furthermore, differences in the scale and nature of cross-border livestock trade with Iraq, compared to the intensive, often unregulated movement with Afghanistan and Pakistan in the east, may result in lower viral introduction pressure. Variations in local livestock husbandry and tick control practices could also contribute to the observed epidemiological pattern (Tavassoli et al., 2024).

### 2.2 Turkey

Turkey has been one of the most affected countries, with over 10,000 cases reported since 2002, predominantly in the Black Sea and Central Anatolia regions. Agricultural practices, such as unregulated livestock farming and seasonal migration of sheep and cattle, have been linked to human exposure (Dinçer et al., 2022). A 2018 study highlighted that 70% of cases occurred among farmers and slaughterhouse workers, underscoring occupational risks (Vatansever, 2018). Despite nationwide awareness campaigns and acaricide use since 2007, Turkey's CFR remains at 5–10%, reflecting challenges in early diagnosis and healthcare access in rural areas (Mehmood et al., 2022).

### 2.3 Iraq

In Iraq, CCHF re-emerged in 2008 after decades of underreporting, with outbreaks concentrated in the southern provinces of Dhi Qar and Basrah, where marshlands provide ideal habitats for *Hyalomma* ticks (Al Salihi et al., 2023). Conflict-related disruptions to healthcare infrastructure and livestock surveillance have exacerbated the crisis. A study documented a CFR of 25% among confirmed cases between 2015 and 2019, with delays in hospitalization and limited ribavirin availability cited as key contributors. Additionally, ritual slaughter practices during Eid al-Adha have been implicated in occupational and community-based outbreaks, highlighting cultural factors in disease spread (Saleh et al., 2025).

### 2.4 Afghanistan and Pakistan

Afghanistan and Pakistan, sharing porous borders with Iran, face overlapping CCHF risks due to refugee movements and unregulated livestock trade. In Afghanistan, sporadic outbreaks in Herat and Kandahar provinces have been linked to returning refugees and displaced populations (Sahak et al., 2019). A 2021 retrospective analysis identified a CFR of 43.3% among hospitalized patients, with limited access to diagnostics and personal protective equipment (PPE) in rural clinics (Qaderi et al., 2021). In Pakistan, Balochistan and Khyber Pakhtunkhwa provinces are endemic hotspots, with outbreaks peaking during the Hajj and Eid seasons due to increased livestock slaughter (Yaqub et al., 2019). A 2020 genomic study revealed that 82% of viral strains in Pakistan clustered with Iranian and Afghan isolates, emphasizing cross-border viral exchange (Umair et al., 2020).

## 3 Regional challenges and future directions

The region's CCHF burden is compounded by climate change, which expands tick habitats, and political instability, which disrupts surveillance (Nili et al., 2020). For instance, rising temperatures in Iran and Turkey have extended *Hyalomma* activity into winter months, increasing year-round transmission risks (Shahbazi et al., 2019). Furthermore, Afghanistan's ongoing conflict has crippled its healthcare system, leaving CCHF cases underreported and untreated (Mustafa et al., 2011).

This systematic review synthesizes two decades of epidemiological data from Iran and its neighboring endemic corridors to elucidate transmission dynamics, risk factors, and gaps in regional preparedness. By integrating findings from Turkey, Iraq, Afghanistan, and Pakistan, this study aims to inform cross-border collaboration and targeted interventions to mitigate CCHF's regional threat.

## 4 Materials and methods

This systematic review adhered to PRISMA guidelines. Articles published between 2005 and 2025 were retrieved from PubMed, Scopus, Web of Science, and Google Scholar using keywords: “Crimean Congo hemorrhagic fever”, “epidemiology”, “Iran”, “Turkey”, “Iraq”, “Afghanistan”, and “Pakistan. Inclusion criteria encompassed English language studies reporting primary epidemiological data on human cases. To provide a comprehensive context for the human epidemiological patterns, studies reporting critical supporting data on virological characteristics (genotypes), primary vectors (*Hyalomma* ticks), and animal reservoirs (seroprevalence in livestock) from the same region and time period were also included. Case reports, reviews, and non-peer reviewed articles were excluded. Study quality was assessed using the STROBE checklist (score ≥ 20/33). Data extraction focused on human case demographics, as well as vectors, reservoirs, viral strains, and CFR to elucidate the interconnected drivers of human disease. We acknowledge that excluding non-English publications and local journals is a limitation, which may introduce selection bias; however, our focus on peer-reviewed international databases ensures consistency in quality and accessibility.

## 5 Results

### 5.1 Epidemiological trends

**Fig. 1:**
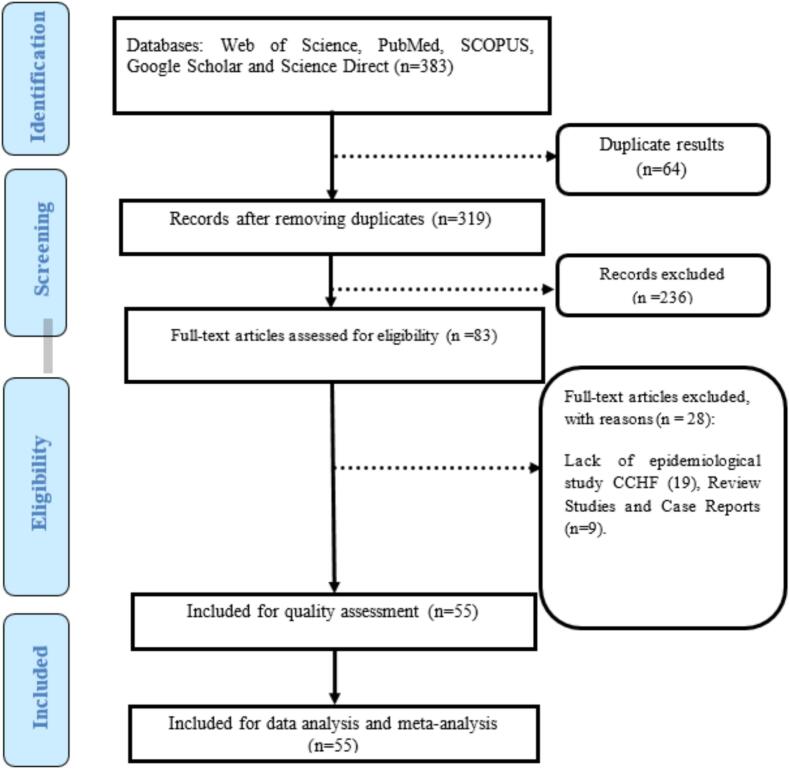
The PRISMA flow diagram.

Of 383 initially identified studies, 55 met inclusion criteria (Fig. 1). Furthermore, Key findings are summarized in Table 1, Table 2.

**Table 1:** Details of several Important CCHF Studies published of Iran During 2005–2025.

Place of study	Year (Reference(s))	Case fatality rate (%)	Amplifying Host(s)	Vector	CCHFV subtype
Khorasan Razavi	2020 (Nili et al., 2020; Maghsood et al., 2020)	8.42	–	*Hyalomma*	Afghanistan and Pakistan strains
Northeast of Iran, and Kurdistan	2019 (Choubdar et al., 2019; Moradi et al., 2019; Saghafipour et al., 2019; Shahbazi et al., 2019)	21.1	Sheep, goat, cattle, and camel, Human	*Hyalomma*	–
Iran	2018 (Ahmadkhani et al., 2018)	–	–	*–*	–
Golestan	2017 (Aslani et al., 2017; Sedaghat et al., 2017)	10	–	*Hyalomma*	–
Border areas of Iran and Pakistan	2017 (Shahhosseini et al., 2017)	–	–	*Hyalomma and Rhipicephalus*	IV-(Asia 1) and IV-(Asia 2)
Golpaygan, Northeast of Iran, and Khuzestan	2016 (Biglari et al., 2016; Sharififard et al., 2016; Farhadpour et al., 2016)	–	–	*Hyalomma dromedarii, Hyalomma anatolicum, Rhipicephalus and Haemaphysalis*	IV-(Asia 1) ، IV-(Europe) and IV-(Asia 2)
Kermanshah	2016 (Mohammadian et al., 2016)	–	Sheep, goat, and cattle	*Hyalomma*	IV-(Asia 1)
Mazandaran	2015 (Faghihi et al., 2015)	–	–	*Rhipicephalus bursa and Hyalomma marginatum*	–
Zahedan	2015 (Sharifinia et al., 2015)	–	Sheep, goat, and cattle	*Hyalomma*	–
Sistan and Baluchestan, Iran	2014 (Mostafavi et al., 2014)	10, 14.5	–	*–*	–
Khorasan	2014 (Champour et al., 2014)		Camel	*–*	–
Iran	2013 (Mostafavi et al., 2013)	–	–	*–*	Russian strains
Sistan and Baluchestan	2013 (Mehravaran et al., 2013)	–	–	*Hyalomma*	–
Qom, Isfahan, Kurdistan, and Fars	2012 (Chinikar et 1al., 2012; Telmadarraiy et al., 2012)	–	Sheep, goat, and cattle	*Hyalomma marginatum‚ Hyalomma anatolicum, Rhipicephalus sanguineus, and Rhipicephalus bursa*	Pakistan, Iraqis, and Afghanistan strains
Chaharmahal and Bakhtiari	2012 (Mahzounieh et al., 2012)	–	–	*–*	Turkey strains
Mazandaran, and Ardabil	2012 (Morovvati et al., 2012; Mostafavi et al., 2012)	–	Sheep, goat, and cattle	-*Hyalomma and Rhipicephalus*	IV-(Asia 1) and IV-(Europe)
Iran	2010 (Ghorbani et al., 2010)	20	–	*–*	Pakistan strains
Sistan and Baluchestan, and Hamedan	2010 (Tahmasebi et al., 2010)	14	–	*Hyalomma*	Pakistan strains
Zahedan	2009 (Shifimoud et al., 2009)	–	–	*–*	Pakistan strains
Sistan and Baluchestan, Isfahan, Tehran and Fars	2008 (Chinikar et al., 2008)	–	–	*–*	–
Southeastern Iran	2006 (Alavi-Naini et al., 2006)	18.75	–	*Hyalomma*	–

**Table 2:** Details of several important CCHF studies published of Iran's neighboring high-risk territories during 2005–2025.

Place of study	Year (References)	Case fatality rate (%)	Amplifying Host(s)	Vector	CCHFV subtype
Pakistan	2020 (Kasi et al., 2020a)	–	Sheep and cattle	–	–
Pakistan	2020 (Kasi et al., 2020b)	–	Sheep, goat, and cattle	*Hyalomma*	IV-(Asia 1)
Pakistan	2017 (Alam et al., 2017)	–	–	–	–
Pakistan	2017 (Halim et al., 2017)	12.8–31	–	–	–
Pakistan	2015 (Abbas et al., 2015)	–	–	–	–
Pakistan	2014 (Hasan et al., 2014)	–	–	–	–
Afghanistan	2019 (Hatami et al., 2019)	15	–	–	–
Afghanistan & Pakistan	2015 (Khurshid et al., 2015)	20.4	Sheep, goat, and cattle	–	IV-(Asia 1)
Afghanistan	2012 (Mofleh and Ahmad, 2012)	33	–	–	–
Afghanistan	2011 (Mustafa et al., 2011)	–	–	*Hyalomma*	–
Afghanistan	2021 (Qaderi et al., 2021)	21.6	–	–	–
Afghanistan	2019 (Sahak et al., 2019)	43.3	–	–	–
Pakistan	2020 (Shahid et al., 2020)	–	–	–	–
Pakistan	2020 (Umair et al., 2020)	40.7	–	*Hyalomma*	IV-(Asia 1)
Pakistan	2020 (Zohaib et al., 2020)	–	Sheep, goat, cattle, and camel	–	Afghanistan and Pakistan strains
Pakistan	2019 (Yaqub et al., 2019)	–	Human	–	IV-(Asia 1)
Turkey	2018 (Vatansever, 2018)	10	Cattle	*Hyalomma anatolicum*	V-(Europe)
Turkey	2022 (Dinçer et al., 2022)	8	Sheep, cattle	*Hyalomma marginatum*	V-(Europe)
Turkey	2015 (Sevinc and Xuan, 2015)	7	Sheep	*Hyalomma* spp.	V-(Europe)
Iraq	2023 (Altaliby et al., 2023)	19.16, 6.25	Sheep, goats	*Hyalomma*	IV-(Asia 1)
Iraq	2024 (Dakhil et al., 2024)	13.23, 4.2	Cattle, goats	*Hyalomma anatolicum*	IV-(Asia 1)
Iraq	2025 (Saleh et al., 2025)	49.3	Sheep and goats	*Hyalomma marginatum*	IV-(Asia 1)

### 5.2 Demographic and occupational risk factors

**Iran:**

• **Gender and Age:** Males accounted for 78–82% of cases, primarily in the 15–44 age group. This aligns with occupational exposure, as men dominate high-risk professions such as livestock herding (41.9%) and slaughterhouse work (Shahhosseini et al., 2017).
• **Case Fatality Rate (CFR):** CFR ranged from 8.42% (Khorasan Razavi) to 21.1% (Sistan and Baluchestan). Higher mortality in southeastern Iran correlated with delayed healthcare access and ribavirin shortages in rural clinics.

***Afghanistan and Pakistan***

• **Gender Disparity:** Similar to Iran, 80–85% of cases occurred in males, with a mean age of 34–37 years. Butchers and healthcare workers constituted 30% of cases in Pakistan's Balochistan province, reflecting both occupational and nosocomial transmission risks (Shahhosseini et al., 2017).
• **CFR Variability:** Afghanistan reported a CFR of 21.6–43.3%, while Pakistan's CFR ranged from 12.8% to 40.7%. The stark contrast highlights disparities in healthcare infrastructure, with Afghanistan's conflict zones showing the poorest outcomes.

The predominance of male cases strongly suggests a higher risk due to occupational hazards in livestock-dependent economies. However, socio-cultural factors leading to differential healthcare-seeking behavior between genders may also influence these reported proportions, potentially resulting in under-detection of cases among women. Higher CFR in border regions (e.g., Sistan and Baluchestan, Balochistan) emphasizes systemic gaps in rural healthcare and diagnostics (Shahhosseini et al., 2017).

### 5.3 Seasonal and geographic patterns

• **Seasonality:** Across all three countries, 74–86% of cases occurred in spring and summer (March–August), coinciding with *Hyalomma* tick activity and livestock breeding cycles.
• **Hotspot Regions:**
o **Iran:** Sistan and Baluchestan (35% of national cases), Golestan, and Kerman.
o **Pakistan:** Balochistan (60% of cases), particularly districts bordering Iran (e.g., Killa Abdullah).
o **Afghanistan:** Herat and Kandahar provinces, linked to cross-border trade with Iran.

The overlap of peak transmission seasons with agricultural activities (e.g., sheep shearing, Eid al-Adha slaughtering) amplifies human exposure. Geographic clustering in border areas underscores the role of unregulated livestock movement in sustaining endemicity.

### 5.4 Vector and host dynamics

• **Vectors:** *Hyalomma* spp. (*H. marginatum*, *H. anatolicum*) were the primary vectors in 88% of studies. Secondary vectors included *Rhipicephalus* (9%) and *Haemaphysalis* (3%).
• **Amplifying Hosts:**
o Domestic ruminants are critical amplifying hosts in the CCHFV transmission cycle. Sheep and goats were implicated in 68% of Iranian studies, cattle in 24%, and camels in 8%.
o In Afghanistan and Pakistan, cattle and sheep accounted for 75% of seropositive animals, with camels playing a lesser role (Qaderi et al., 2021).

The presence of these animals, particularly during periods of viremia, is a significant risk factor for human infection, both via tick bites that have fed on infected animals and through direct contact during slaughter or husbandry.

### 5.5 Virological characteristics

• **Genotypes:**
o **Iran:** Asia 1 (Clade IV) predominated (80%), with minor circulation of Europe (Clade V, 14%) and Asia 2 (Clade IV, 6%) strains. Phylogenetic analysis revealed 99.4% similarity between Iranian and Pakistani strains.
o **Afghanistan/Pakistan:** Asia 1 accounted for 85–90% of sequenced isolates, with sporadic detection of Asia 2 in Pakistani border regions.

The dominance of Asia 1 across all three countries suggests a shared evolutionary origin, likely facilitated by cross-border livestock trade. The presence of Europe clade strains in Iran may indicate historical introductions from Turkey or Eastern Europe.

### 5.6 Cross-border transmission

• **Informal Livestock Movement:** The transboundary spread of CCHFV is facilitated by informal livestock trade and seasonal pastoral migration, often occurring over local and regional distances rather than continent-spanning routes. For instance, 62% of CCHFV-positive livestock in Pakistan's Balochistan were sourced from Afghanistan, while 25% of Iranian cases in Sistan and Baluchestan involved Afghan migrants, many of whom work with locally traded animals (Tavassoli et al., 2024).

These movements primarily connect adjacent border provinces (e.g., Herat in Afghanistan with Sistan and Baluchestan in Iran; Kandahar with Balochistan in Pakistan), creating interconnected local transmission zones that collectively form a larger regional network.

• **Human Mobility and Infection Origin:**
  A significant proportion (25%) of Iranian cases in Sistan and Baluchestan involved Afghan migrants. It is important to note that these individuals could have been infected in their country of origin prior to migration or acquired the infection within Iran while working in high-risk occupations like livestock handling.
• **Phylogenetic Evidence:** Viral strain clustering confirms cross-border exchange but does not specify directionality. Strains from Iran's Khorasan Razavi clustered with Afghan isolates (Accession: GU456725.1), while Balochistan (Pakistan) strains matched those from southeastern Iran (Accession: AY366378.1).

Unregulated livestock movement and migration are critical drivers of sustained viral circulation across borders. Viral strain clustering confirms that border regions act as conduits for bidirectional pathogen exchange, necessitating binational surveillance.

## 6 Discussion

The epidemiological landscape of Crimean-Congo hemorrhagic fever (CCHF) in Iran and its neighboring countries is shaped by a complex interplay of ecological, socioeconomic, and geopolitical factors. This review highlights the interconnectedness of transmission dynamics across this region, where shared vectors, amplifying hosts, and human activities perpetuate endemicity. While previous reviews have documented CCHF epidemiology in individual countries, our analysis uniquely synthesizes evidence to illustrate how Iran serves as an epidemiological crossroads, with cross-border movements and shared viral strains sustaining transmission across the region. Below, we contextualize these findings within regional frameworks, emphasizing Iran's role as a nexus for cross-border transmission and comparing country-specific challenges and interventions.

### 6.1 Iran: a regional epicenter and crossroads for transmission

Iran's status as a CCHF hotspot is inextricably linked to its geographic position, bordering Afghanistan, Pakistan, Iraq, and Turkey—all endemic countries. Provinces such as Sistan and Baluchestan, Golestan, and Kerman, which account for 65% of national cases, are part of larger, shared endemic foci, where unregulated livestock trade and seasonal migration perpetuate a continuous cycle of transboundary pathogen exchange (Alavi-Naini et al., 2006; Ahmadkhani et al., 2018). The predominance of the Asia 1 genotype (Clade IV) in Iran, which shares 99.4% genetic similarity with strains from Afghanistan and Pakistan, underscores the permeability of borders to viral spread (Mustafa et al., 2011).

Iran's eastern regions, particularly Sistan and Baluchestan, face unique challenges. The influx of Afghan migrants, many employed in high-risk occupations, amplifies exposure. These migrant workers are a vulnerable population who may have been infected in Afghanistan or who face high exposure risk within Iran, thus acting as a potential bridge for the virus in both directions across the border. Furthermore, acaricide resistance in *Hyalomma* populations and fragmented healthcare access in rural areas exacerbate CFR (14.5%). Despite Iran's early adoption of national surveillance in 2000, political tensions and resource limitations in border zones hinder comprehensive reporting, leaving gaps in outbreak preparedness.

The variability in CCHF virus across the region, particularly the higher rates in southeastern Iran, eastern Afghanistan, and western Pakistan, cannot be dissociated from disparities in healthcare infrastructure. The debated but potential early-stage efficacy of ribavirin underscores the critical importance of timely medical intervention. In conflict-affected and remote pastoral communities, barriers to healthcare access—including distance to clinics, cost of transport, and shortages of medical supplies—result in significant delays. Patients often present late in the clinical course, diminishing the potential effectiveness of supportive care and any antiviral treatment, thereby exacerbating CFR. This systemic challenge highlights the need for decentralized healthcare services, stockpiling of essential medicines in rural clinics, and community-based education to encourage early seeking of care following potential exposure.

### 6.2 Turkey: successes and persistent gaps in control

Turkey, reporting over 10,000 cases since 2002, exemplifies both progress and unresolved challenges in CCHF management. The Black Sea and Central Anatolia regions, where small-scale farming dominates, remain epicenters due to occupational exposure. However, Turkey's CFR (5–10%) is notably lower than Iran's or Afghanistan's, attributable to nationwide awareness campaigns, early ribavirin deployment, and improved diagnostics post-2007 (Mehmood et al., 2022).

Turkey's experience offers critical lessons. For instance, mandatory PPE use among slaughterhouse workers and subsidized acaricides for livestock have reduced nosocomial and tick-borne transmission. Yet, rural healthcare disparities persist, with delayed hospitalization contributing to preventable deaths. Additionally, climate change is contributing to the expansion of *Hyalomma* tick habitats and activity periods, challenging traditional seasonal control strategies (Shahbazi et al., 2019).

### 6.3 Iraq: conflict, ecology, and resurgent outbreaks

Iraq's CCHF burden, concentrated in the marshlands of Dhi Qar and Basrah, reflects the intersection of ecological fragility and political instability. The re-emergence of CCHF in 2008 after decades of quiescence correlates with post-war disruptions to veterinary surveillance and healthcare infrastructure. Marshland drainage projects, aimed at agricultural expansion, have inadvertently created ideal habitats for *Hyalomma* ticks, while ritual slaughter practices during Eid al-Adha facilitate nosocomial outbreaks (Al Salihi et al., 2023).

Iraq's CFR (25%)—higher than Turkey's but lower than Afghanistan's—highlights moderate healthcare capacity but underscores the urgency of rebuilding post-conflict systems. International aid programs focusing on tick control and community education in southern Iraq could mitigate risks, though security concerns complicate implementation (Saleh et al., 2025).

### 6.4 Afghanistan and Pakistan: conflict zones and uncontrolled spread

Afghanistan and Pakistan represent the most volatile CCHF landscapes, where conflict and displacement intersect with endemic transmission. In Afghanistan, Herat and Kandahar provinces report CFRs as high as 43.3%, driven by diagnostic shortages, inadequate PPE, and a collapsed healthcare system (Sahak et al., 2019). Returning refugees and internally displaced populations, often residing near livestock, further propagate outbreaks (Qaderi et al., 2021).

Pakistan's Balochistan province, sharing a porous border with Iran and Afghanistan, mirrors these challenges. Here, 60% of cases are linked to livestock imported from Afghanistan, and phylogenetic studies confirm viral strain overlap with Iranian isolates (Yaqub et al., 2019). Seasonal peaks during Eid al-Adha and Hajj, marked by mass livestock slaughter, highlight cultural practices as amplifiers of zoonotic risk. Despite Pakistan's advanced genomic surveillance capacity, political instability in Balochistan limits the reach of public health interventions (Umair et al., 2020).

### 6.5 Beyond immediate neighbors: the broader regional context

While this review focuses on Iran's contiguous neighbors, CCHF risks extend to other countries in the surrounding region. For instance:

• **Armenia and Azerbaijan**: Limited data suggest sporadic CCHF cases linked to transhumance practices along the Iranian border, though underreporting obscures true incidence.
• **Turkmenistan**: Shared ecosystems with Iran's Golestan province likely support *Hyalomma* habitats, but political isolation restricts data sharing and collaborative surveillance.
• **Saudi Arabia**: Though non-endemic, Hajj pilgrimages from Pakistan and Afghanistan pose importation risks, necessitating screening protocols at entry points.

The absence of robust data from these regions underscores a critical gap in a comprehensive regional risk assessment.

### 6.6 Socioeconomic and ecological drivers of transmission

#### 6.6.1 Pastoral economies and occupational hazards

Across the studied countries, pastoralism remains a cornerstone of rural livelihoods, perpetuating human-animal contact. In Iran, 41.9% of cases occur among ranchers and butchers, while in Afghanistan, returning refugees often resort to livestock work due to limited economic alternatives (Champour et al., 2014; Morovvati et al., 2012). Gender disparities in reported cases—with men constituting 78–85% of cases—reflect cultural norms assigning high-risk labor to males (Shahhosseini et al., 2017). It is important to consider that these figures may be influenced by gendered patterns in healthcare access and attendance, where men in these communities might be more likely to seek and receive a formal diagnosis, potentially leading to an under-ascertainment of cases in women.

#### 6.6.2 Climate change and expanding tick habitats

Rising temperatures and altered precipitation patterns are extending *Hyalomma* activity periods and geographic ranges. In Iran and Turkey, warmer winters have enabled year-round tick proliferation, while marshland degradation in Iraq has created new niches for vectors (Dakhil et al., 2024). Climate models predict further habitat expansion into currently non-endemic areas, necessitating adaptive surveillance strategies.

#### 6.6.3 Cultural and religious practices

Religious festivals like Eid al-Adha, which involve mass livestock slaughter, are double-edged swords. While culturally significant, they concentrate high-risk activities, as seen in Pakistan's Balochistan and Iran's Sistan and Baluchestan (Mostafavi et al., 2013). Similarly, traditional healing practices in rural Afghanistan delay hospital referrals, increasing CFRs (Sahak et al., 2019).

### 6.7 Towards a regional one health framework

The genetic homogeneity of CCHFV strains across borders, particularly the dominance of Asia 1, underscores that isolated national responses are often insufficient for long-term control, as they can be undermined by cross-border viral exchange. However, as the reviewer rightly points out, robust national prevention programmes targeting at-risk populations are crucial and can significantly reduce exposure risk within endemic areas. Furthermore, the role of wild animals in maintaining CCHFV enzootic cycles in the region remains poorly understood and represents a critical knowledge gap. A regional One Health approach, integrating human, animal, and environmental health, is therefore imperative to complement national efforts.

Key strategies include:

1. **Standardized Surveillance**: Harmonizing case definitions and reporting protocols across countries to enable real-time data sharing.
2. **Cross-Border Tick Control**: Coordinated acaricide campaigns targeting migratory livestock routes, particularly in Sistan-Baluchestan (Iran) and Balochistan (Pakistan).
3. **Community Engagement**: Culturally tailored education programs to mitigate risks during festivals and promote PPE use among high-risk groups.
4. **Conflict-Zone Interventions**: Partnering with NGOs to deploy mobile clinics and rapid diagnostics in Afghanistan and Iraq.
5. **Climate Adaptation**: Monitoring tick habitat shifts and integrating climate forecasts into outbreak preparedness.

### 6.8 Limitations and future directions

This review's reliance on published data introduces biases, particularly in conflict zones like Afghanistan, where underreporting is rampant. Furthermore, the observed gender disparity in cases is likely confounded by differential healthcare-seeking behaviors and access for men and women in the endemic regions, which could lead to under-detection and under-reporting of cases among women. Additionally, the exclusion of non-English studies may overlook regional insights. While previous reviews have provided broad regional overviews, our focused analysis on Iran's role as a transmission nexus reveals specific gaps in cross-border surveillance and control that warrant urgent attention. Furthermore, the studies reviewed seldom differentiated between human cases acquired through direct tick bites versus those resulting from contact with the blood of viraxic amplifying hosts. This lack of granular data limits our ability to precisely quantify the relative importance of these two transmission routes in different ecological and occupational settings.

Future research should prioritize:

• **Genomic Surveillance**: Tracking emerging strains and acaricide resistance markers.
• **Socioeconomic Studies**: Evaluating the impact of poverty and displacement on exposure risks.
• **Regional Collaboratives**: Establishing platforms like the Middle East CCHF Consortium to pool resources and expertise.

## 7 Conclusion

CCHF in Iran and its neighboring countries is not merely a biomedical issue but a reflection of broader regional complexities—ecological fragility, economic dependency on livestock, and geopolitical instability. While Iran's experience offers a blueprint for surveillance, Turkey's successes in CFR reduction highlight the value of public health investment. Conversely, Afghanistan and Iraq illustrate the catastrophic consequences of neglected healthcare systems. This review moves beyond country-specific accounts to demonstrate how interconnected these epidemiological landscapes are, providing a new perspective on CCHF control that emphasizes regional cooperation over isolated national responses. Addressing CCHF's regional threat demands transcending borders through collaborative science, equitable resource allocation, and a commitment to One Health principles. Only then can this region transition from a hotspot of disease to a hub of resilience.

## CRediT authorship contribution statement

**Jalal Mohammadi:** Writing – review & editing, Formal analysis, Data curation. **Kourosh Azizi:** Writing – review & editing, Supervision, Methodology, Conceptualization. **Zahra Nasiri:** Writing – review & editing, Methodology, Formal analysis. **Mohsen Kalantari:** Writing – review & editing, Writing – original draft, Validation, Methodology, Conceptualization.

## Funding

No funding.

## Declaration of competing interest

The authors declare that they have no known competing financial interests or personal relationships that could have appeared to influence the work reported in this paper.

## Data Availability

Data will be made available on request to M.K.

## Footnotes

1 *In this review, the term “broader Middle East/West Asia region” is used to denote the high-risk zone encompassing Iran, Turkey, Iraq, Afghanistan, and Pakistan, acknowledging the interconnected nature of CCHF transmission despite varying geographical definitions.*
